# PtyNAMi: ptychographic nano-analytical microscope^1^

**DOI:** 10.1107/S1600576720008420

**Published:** 2020-07-30

**Authors:** Andreas Schropp, Ralph Döhrmann, Stephan Botta, Dennis Brückner, Maik Kahnt, Mikhail Lyubomirskiy, Christina Ossig, Maria Scholz, Martin Seyrich, Michael E. Stuckelberger, Patrik Wiljes, Felix Wittwer, Jan Garrevoet, Gerald Falkenberg, Yakub Fam, Thomas L. Sheppard, Jan-Dierk Grunwaldt, Christian G. Schroer

**Affiliations:** a Deutsches Elektronen-Synchrotron DESY, Notkestrasse 85, DE-22607 Hamburg, Germany; b Department Physik, Universität Hamburg, Luruper Chaussee 149, DE-22761 Hamburg, Germany; c MAX IV Laboratory, Fotongatan 2, SE-225 94 Lund, Sweden; d Institute for Chemical Technology and Polymer Chemistry, Karlsruhe Institute of Technology, Engesserstrasse 20, DE-76131 Karlsruhe, Germany; e Institute of Catalysis Research and Technology, Karlsruhe Institute of Technology, Hermann-von-Helmholtz Platz 1, DE-76344 Eggenstein-Leopoldshafen, Germany

**Keywords:** ptychography, multi-modal imaging, X-ray imaging, *in situ* imaging, tomography

## Abstract

Ptychographic X-ray imaging at the highest spatial resolution requires an optimal experimental environment, providing a high coherent flux, excellent mechanical stability and a low background in the measured data. This requires, for example, a stable performance of all optical components along the entire beam path, high temperature stability, a robust sample and optics tracking system, and a scatter-free environment. This contribution summarizes the efforts along these lines to transform the nanoprobe station on beamline P06 (PETRA III) into the ptychographic nano-analytical microscope (PtyNAMi).

## Abstract 2

The ptychographic nano-analytical microscope (PtyNAMi) is designed for *in situ*/*operando* high-resolution imaging in two and three dimensions with high sensitivity and structural, elemental, chemical and electronic contrast.

## 1. Introduction   

Understanding the structure and function of matter on the microscopic scale is of utmost importance to develop new functional materials or understand complex processes in nature and technology. Ideally, studies to this end are carried out under *in situ* or *operando* conditions in order to image the sample evolving in its natural environment. While electron microscopy can push the spatial resolution to the extreme in very small and carefully prepared samples, hard X-rays can penetrate thicker samples in a larger and more realistic environment. X-rays give access to local structural, electrical, elemental or chemical information from the inside of even thick samples with high spatial resolution. Hard X-ray microscopy is therefore a powerful method for structure determination in three dimensions, and it is applied in a variety of scientific fields, such as biology, chemistry (including catalysis), physics, materials science and nanotechnology.

The hard X-ray microscopy setup on the PETRA III beamline P06 provides access to different contrast mechanisms such as X-ray absorption spectroscopy, X-ray fluorescence, X-ray beam-induced current and X-ray diffraction (Schroer *et al.*, 2010 ▸). It is designed to produce focused hard X-ray beams with sizes of 50 nm (FWHM) and below. Furthermore, the standard scanning techniques, which are typically limited in spatial resolution by the X-ray focus size, can be combined with scanning coherent X-ray diffraction microscopy (ptychography) (Rodenburg & Faulkner, 2004 ▸; Faulkner & Rodenburg, 2004 ▸; Rodenburg *et al.*, 2007 ▸; Thibault *et al.*, 2008 ▸; Pfeiffer, 2018 ▸), potentially yielding structural information in three dimensions with an even higher spatial resolution of 10 nm and below (Schropp *et al.*, 2012 ▸). Since ptychography is based on coherent diffraction, it is in principle limited in performance only by the available coherent photon flux, rather than the focus size (Schropp & Schroer, 2010 ▸) or the radiation hardness of the sample.

Many practical aspects of such an X-ray microscope hinder the optimal performance, and further instrumental developments were needed in order to reach a resolution regime below 10 nm in X-ray imaging on a routine basis. Our efforts to reach this goal cumulated in an upgraded nanoprobe setup on beamline P06, denoted ptychographic nano-analytical microsocope (PtyNAMi).

The setup is optimized in order to enable scanning hard X-ray microscopy with the highest possible spatial resolution and sensitivity using the aforementioned X-ray analytical contrast mechanisms, and it provides all degrees of freedom required for 2D and 3D imaging experiments (Kahnt *et al.*, 2019 ▸). It is furthermore compatible with additional auxiliary experimental equipment required for *in situ* or *operando* experiments (Fam *et al.*, 2019 ▸). The instrumental design was developed following the experimental requirements for high-resolution X-ray ptychography, which may be summarized as

(i) high-performance X-ray optics (Section 4),

(ii) high mechanical stability and control (Section 5),

(iii) low scattering background (Section 6),

(iv) optimized coherent flux (aperture matching).

These instrumental developments, requiring both a high mechanical and temperature stability and positioning control, are strongly driven by the upcoming synchrotron radiation sources of the fourth generation, such as MAX IV (Tavares *et al.*, 2018 ▸), ESRF-EBS (Raimondi, 2016 ▸) or PETRA IV (Schroer *et al.*, 2018 ▸; Schroer, Röhlsberger *et al.*, 2019 ▸). These new ultralow-emittance X-ray sources will provide a considerably higher coherent photon flux optimal for hard X-ray microscopy. Various microscopy setups are currently being developed at many synchrotron radiation sources worldwide in order to finally close the current resolution gap in X-ray imaging between about 10 nm and the atomic scale (da Silva *et al.*, 2019 ▸; Martinez-Criado *et al.*, 2016 ▸; Nazaretski *et al.*, 2017 ▸; Holler *et al.*, 2018 ▸; Deng *et al.*, 2019 ▸; Takahashi *et al.*, 2011 ▸).

This article is structured mainly in three parts, starting with a general introduction to beamline P06 (Section 2). This is followed by a detailed description of instrumental developments implemented on PtyNAMi and the specific performance characteristics of the setup (Sections 3 [Sec sec4] [Sec sec5] to 6). Finally, some representative high-resolution nano-imaging results obtained with the new setup are shown (Section 7).

## 2. Beamline P06 at PETRA III   

**Figure 1:**
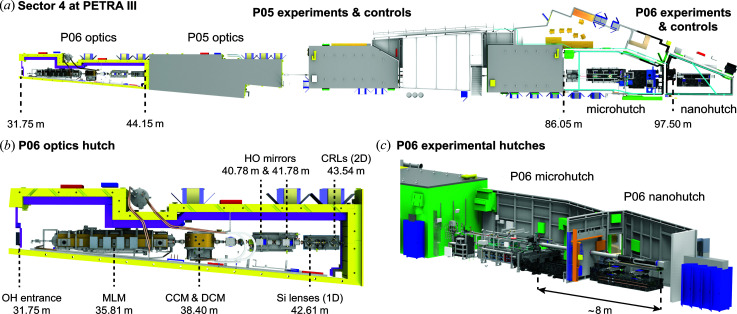
(*a*) An overview of Sector 4 at PETRA III. Beamline P06 shares this sector of the storage ring with the imaging beamline P05. (*b*) The P06 optics hutch comprises a multilayer monochromator (MLM), a channel-cut crystal monochromator (CCM), a double-crystal monochromator (DCM), horizontal offset mirrors (HO mirrors), and 1D refractive silicon lenses (1D lenses) and beryllium compound refractive lenses (CRLs) for prefocusing. Indicated position values refer to the distance from the middle of the undulator to the different devices. (*c*) A CAD drawing of the experimental hutches on beamline P06.

The ptychographic nano-analytical microscope (PtyNAMi) is installed in the nanohutch of beamline P06, the hard X-ray micro/nanoprobe at the synchrotron radiation source PETRA III (DESY, Hamburg). The storage ring is characterized by its low emittance of 1.3 × 0.013 nm rad in the horizontal (h) and vertical (v) directions, respectively. Beamline P06 shares a low-β section (Sector 4) of the storage ring with the imaging beamline P05. The X-ray light on P06 is produced by a 2 m long U32 undulator (λ_U_ = 31.4 mm and *K*_max_ = 2.7), creating a small X-ray source with a size of about 36 µm (h) × 6.9 µm (v) (r.m.s. at *E* = 12 keV) and a source divergence of 28 µrad (h) × 4 µrad (v) (r.m.s. at *E* = 12 keV). A schematic layout of beamline P06 is shown in Fig. 1 ▸ [see also Table 1 ▸ for specific distance values and a description of the previous nanoprobe setup on P06 (Schroer *et al.*, 2016 ▸)].

### 2.1. P06 optics hutch   

The optics hutch on beamline P06 [see Fig. 1 ▸(*b*)] lies between 31.8 and 44.15 m from the X-ray source. It contains a two-bounce multilayer monochromator (MLM) with a bandwidth of a few percent, a cryogenically cooled double-crystal monochromator (DCM) with a fixed vertical offset and a channel-cut crystal monochromator (CCM). Both crystal monochromators are equipped with Si(111) crystals. Horizontal-offset mirrors (HO mirrors) with Si, Cr and Pt coatings suppress higher harmonics of the X-ray beam with cutoff energies between 6 and 30 keV. Prefocusing X-ray lenses at the end of the optics hutch can be used to adapt the photon flux or the coherence properties of the X-ray beam to the optics of the X-ray microscopes in both the micro- and nanohutch (aperture matching) (Schroer *et al.*, 2010 ▸; Schroer & Falkenberg, 2014 ▸).

Recently, 1D nanofocusing lenses made out of silicon were implemented in an additional small vacuum chamber in order to prefocus the X-ray beam in the vertical direction, thereby collecting a larger coherent fraction of the X-ray beam [see Fig. 1 ▸(*b*)]. The X-ray energy can be continuously tuned between 6 and 18 keV or 2.4 and 50 keV using the channel-cut or the double-crystal monochromator, respectively. The multilayer monochromator offers an energy range between 10 and 100 keV.

In addition, beamline P06 is equipped with various slit systems and beam monitors along the X-ray beam path. The high-power slits PS1 and PS2 are located still within the PETRA III ring tunnel, while a quadrant beam-position monitor is positioned about 1 m behind the DCM and followed at a close distance by a retractable beam monitor (LM2) in the optics hutch. Another beam monitor (LM2b) is installed directly behind the new 1D focusing system, facilitating the alignment of the 1D silicon lenses and HO mirrors. A slit system in the optics hutch (OH slits) is used to further define the X-ray beam before it passes through the 2D prefocusing system based on beryllium compound refractive lenses (Be CRLs) (Lengeler *et al.*, 1999 ▸). The CRL pre­focusing system can accommodate a maximum of six cartridges, each containing a different combination of Be CRL stacks. Details of the specific CRL assemblies in the different cartridges are summarized in Table 2 ▸.

The current prefocusing configuration is equivalent to a system of lens stacks containing integer binary exponentials of Be CRLs with a radius of curvature of *R* = 1.5 mm, except for the last one which is slightly more focusing. In particular, the six cartridges contain an equivalent of *N* = 2, *N* = 4, *N* = 8, *N* = 16, *N* = 32 and *N* = 67.5 lenses with *R* = 1.5 mm, respectively. The first cartridges are typically used to maximize the photon flux, whereas the last, more strongly focusing, ones are inserted in order to enhance the spatial coherence at the microscope by creating a virtual source between the optics and the experimental hutch. Each cartridge can be inserted independently into the X-ray beam and therefore a variety of different lens configurations are possible. In this way, the monochromatic beam can be optimally shaped over a wide X-ray energy range, depending on the specific experimental needs at the position of the X-ray microscopes in either the micro- or the nanohutch.

### 2.2. P06 microhutch   

The next beam monitor (LM3) is positioned closely downstream of the optics hutch at a distance of 44.66 m from the undulator source. From there, the X-ray beam can propagate freely until it enters the P06 microhutch at a distance of 86.05 m from the source (see Fig. 1 ▸). At the beginning of the microhutch another beam monitor (LM4), followed by a second quadrant beam-position monitor (QBPM micro), are used to measure the position and direction of the X-ray beam. Just downstream of these components, a fast shutter (Azsol SLS 200) is implemented to control the exposure time precisely. It is followed by an absorber box containing 12 foils (three silicon wafers with thicknesses from 380 to 3000 µm and nine aluminium foils with thicknesses from 5 to 2000 ▸ µm) in order to attenuate the X-ray beam if this is required experimentally (see the position details in Table 1 ▸).

This first experimental hutch on beamline P06 comprises the microprobe setup, a hard X-ray scanning microscope based on Kirkpatrick–Baez (KB) mirrors as the main focusing system (Kirkpatrick & Baez, 1948 ▸; Mimura *et al.*, 2007 ▸). Here, X-ray microbeams with a size below 500 nm (FWHM) are routinely used (Boesenberg *et al.*, 2016 ▸; Rumancev *et al.*, 2020 ▸). Recently, Be CRLs in combination with corrective phase plates were also implemented as alternative X-ray nano­focusing optics. By using this setup, the coherent part of the X-ray beam can be focused to a size of about 100 nm (FWHM) (Seiboth *et al.*, 2017 ▸; Schropp *et al.*, 2018 ▸), and it is often used if the beamline is operated in combined mode using both the micro- and nanohutches [see Fig. 1 ▸(*c*)]. In this experimental scenario the full space available on P06 is used, with a distance of up to approximately 8 m between the sample (located in the microhutch) and the diffraction detector (positioned at the end of the nanohutch).

The beamline extends further to the nanohutch, starting at about 96.2 m from the undulator source. This second experimental hutch on beamline P06 provides a high temperature stability of about 0.1 K in order to minimize the influence of thermal drifts. Further experimental or technical equipment, which can potentially act as a heat or vibration source, is located outside the hutch. PtyNAMi is installed in this highly stable environment.

## 3. PtyNAMi   

At the beginning of the nanohutch, an ionization chamber and a 2D X-ray detector (X-ray eye) are positioned at distances of about 115 and 50 mm in front of the entrance slits of the microscope in order to optimize the intensity and visualize the X-ray beam before it enters the instrument, respectively. The entrance slit system (PI miCos GmbH) with integrated pairs of high-precision slit blades (Advanced Design Consulting USA Inc.) is used to confine the incident X-ray beam size further. A transmission diode is implemented a short distance behind the slit system in order to determine the incoming photon flux before the X-ray beam hits the nanofocusing X-ray optics. At a short distance in front of the nanofocus, a pinhole is implemented to clean the X-ray beam from parasitic scattering created within the optics or other optical components upstream. The sample is then positioned close to the focal plane of the optics. Table 3 ▸ summarizes some position values of these components for a typical lens configuration using nanofocusing lenses (NFLs; Schroer *et al.*, 2003 ▸, 2005 ▸) with a working distance of 30 mm. Depending on the particular focusing requirements, these values may vary for other lens configurations or different nanofocusing X-ray optics.

**Figure 2:**
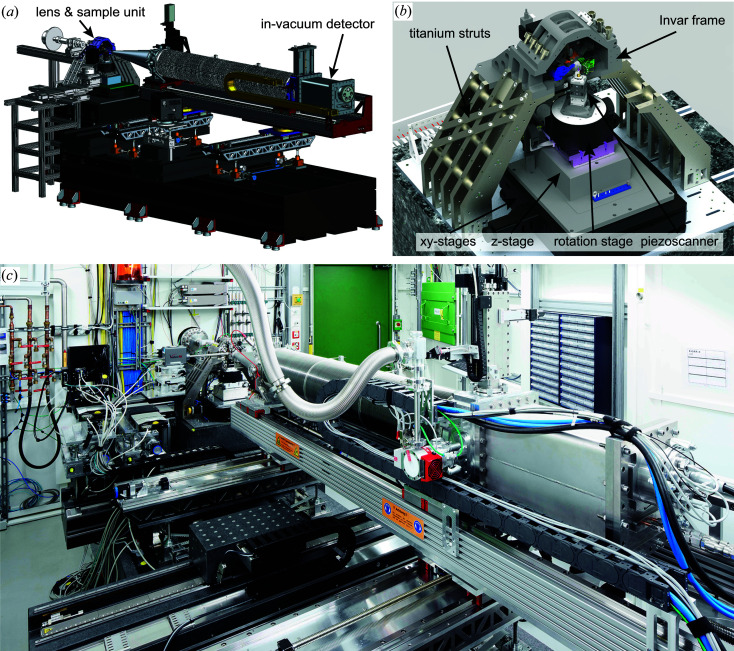
(*a*) A CAD drawing of PtyNAMi, including the lens and sample unit and the detector device. (*b*) The main part (scanner) of the microscope, including the lens and sample unit. (*c*) A photograph showing the current state of PtyNAMi.

In Fig. 2 ▸ an overview of different components of the setup is given. It is mainly divided into two parts, namely the lens and sample platform, and a detector device [see Fig. 2 ▸(*a*)]. The core of the microscope holding the X-ray lenses is built on a stiff and stable titanium and Invar frame [see Fig. 2 ▸(*b*)], which forms the base on which to mount the various X-ray optics such as NFLs, Fresnel zone plates (FZPs) or, recently, multilayer Laue lenses (MLLs). The accurate alignment of these optics requires up to 2 × 6 degrees of freedom, which was realized by implementing two hexapods (SmarAct GmbH), both hanging at an angle of 45° from the top of the scanner frame. In the case of cylindrical optics that focus in one dimension only, each of them carries a lens for either horizontal or vertical X-ray focusing. The sample mount is realized by a stack of linear stages for coarse alignment in the horizontal (CS-430, PI miCos GmbH) and vertical direction (NPE-200, PI miCos GmbH). On top of these, an air-bearing rotation stage (UPR-160, PI miCos GmbH) is installed, allowing rotation of the sample over 360° around the vertical axis. If the interferometric position control using the glass ball retroreflector is active, an angular range of more than 180° can be accessed (see Fig. 4). The fine positioning is then further implemented using a piezo scanner (QNP40-100, Aerotech Inc.), allowing the user to scan samples with nanometre accuracy over a maximum travel range of 100 µm in the *x*, *y* and *z* directions. In addition, two linear centring stages (Q-545, PI miCos GmbH) are used below the scanner to align a sample to the axis of rotation, which is required for tomographic applications. Although the centring stages are not absolutely required, since alignment errors in tomography could also be compensated using the linear stages below the rotation stage, they typically facilitate the sample alignment procedure considerably. Future stability-enhanced upgrades might not consider the use of centring stages in order to further stiffen the setup.

**Figure 3:**
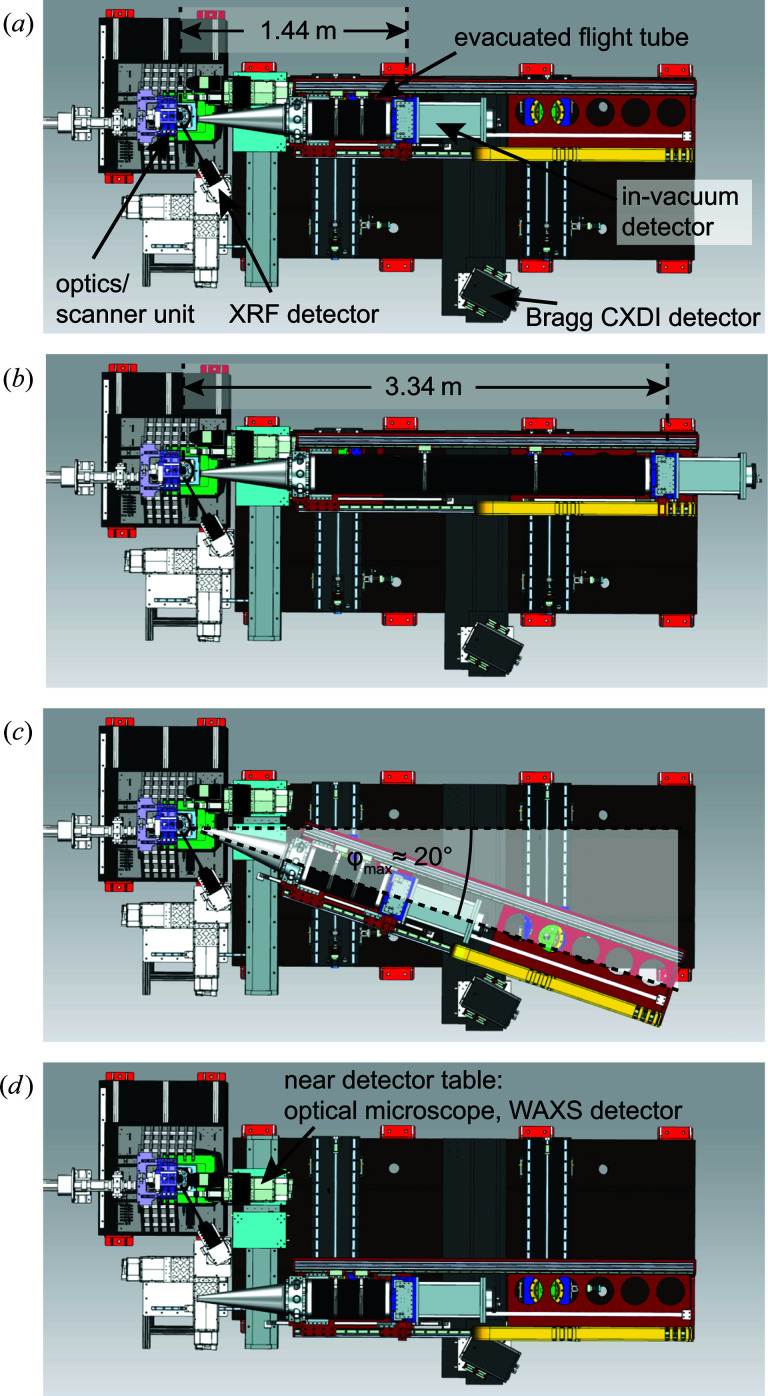
The variable detector configuration (top views). (*a*), (*b*) A flexible bellow allows one to adjust the distance between the sample and in-vacuum detector in a range from 1.44 to 3.34 m. (*c*) The evacuated tube can be rotated around the sample position in the horizontal plane by approximately 20° maximum. (*d*) The near-detector configuration.

The detector device is optimized to support an in-vacuum diffraction detector (Eiger X 4M, Dectris Ltd). The entire detector setup is fully motorized, allowing the user to switch easily between different detector configurations. In Fig. 3 ▸ different geometric scenarios are summarized, showing that the distance between the sample and the in-vacuum diffraction detector can be varied continuously from 1.44 to 3.34 m and the evacuated tube can be swivelled with respect to the direct beam by a maximum angle of φ_max_ ≃ 20° [see Figs. 3 ▸(*a*)–3 ▸(*c*)]. The different components of the detector device, such as the nozzle of the evacuated tube pointing towards the sample and the small vacuum chamber containing the detector at the end of the tube, can be moved independently, providing maximum flexibility in setting up a specific detector geometry.

If required, the evacuated tube can be completely retracted from the X-ray beam path, opening up space for an optical microscope or any additional 2D X-ray detectors which need to be positioned in the vicinity of the sample. While the optical microscope is typically used to align a sample visually, near X-ray detectors are employed for wide-angle X-ray scattering applications [see Fig. 3 ▸(*d*)]. For high-resolution wide-angle scattering applications in the intermediate angular regime between about 20 and 45°, an additional platform is available outside the vacuum to mount another 2D X-ray detector. In this way, Bragg coherent diffraction imaging experiments can be carried out [see Fig. 3 ▸(*a*)] (Stankevič *et al.*, 2015 ▸; Dzhigaev *et al.*, 2016 ▸; Hruszkewycz *et al.*, 2017 ▸). The detector suite is complemented by an X-ray fluorescence detector (Vortex-EM, Hitachi Ltd) collecting the emitted X-ray fluorescence light, typically under an angle close to 90° relative to the incident beam in the horizontal plane [see Fig. 3 ▸(*a*)].

**Figure 4:**
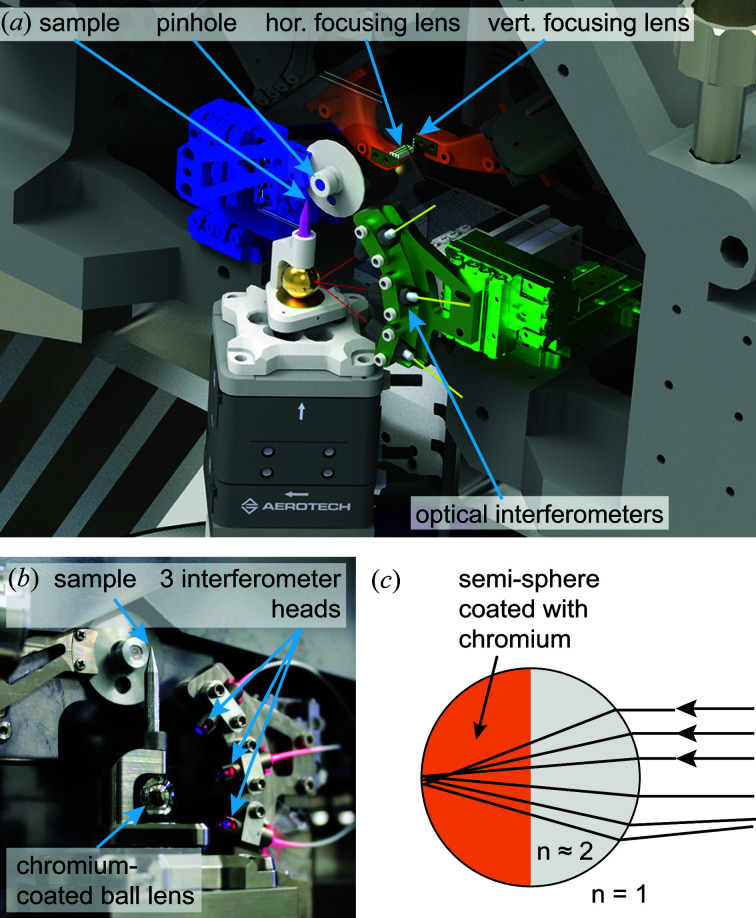
(*a*) A CAD drawing of the PtyNAMi scanner unit, including the X-ray lenses, pinhole, kinematic sample mount and optical interferometers. (*b*) A photograph showing the sample holder with an integrated ball lens as retroreflector. The three interferometer heads point towards the ball lens and can be aligned using a stack of three SmarAct stages. (*c*) A sketch of the working principle of a transparent ball lens acting as a retroreflector if its optical refractive index *n* is close to 2.

In Fig. 4 ▸ the core part of the microscope is shown. It consists of the X-ray lenses for horizontal and vertical focusing, each mounted on a hexapod providing six degrees of freedom for alignment, a pinhole downstream of the lenses, and the sample scanner. The sample is mounted on the scanner with a kinematic mount that includes a ball lens retroreflector whose position is monitored by three interferometer heads mounted in the plane perpendicular to the X-ray beam at angles of approximately −15, 15 and 45° (Schroer *et al.*, 2017 ▸). They point towards the ball lens retroreflector, measuring the position of the sample at a sampling frequency of up to 156 kHz. With this device, the position and mechanical vibrations of the sample can be measured with high accuracy in the plane perpendicular to the X-ray beam. However, the sampling frequencies typically used are smaller than 20 kHz, which is sufficient to cover the main vibrational modes of the setup. The noise level of the interferometers is specified to well below 1 nm, regardless of the streaming frequency. To date, the interferometers are only employed to measure the sample position at high sampling frequencies, but they could in principle also be used for closed-loop positioning control of the sample. The sample motion along the beam axis is not monitored externally, as the depth of focus of the X-ray optical system is typically much larger than all deviations from the nominal position along the optical axis. The interferometer and pinhole holder can be aligned with linear piezo stages (SLC-series, SmarAct GmbH).

The ball lens retroreflector is made out of glass with a refractive index of *n* ≃ 2 (Edmund Optics) and has a diameter of 10 mm. Sample positioning works reliably as long as the sample position stays within a range of about 10% of the sphere’s diameter, *i.e.* about 1 mm in all dimensions. The collimated interferometer beam has a size of about 400 µm. According to the manufacturer, shape errors of the sphere are below 2 µm. Half of the sphere is coated with chromium to enhance the retroreflected signal. Using this device for retroreflection has the main advantage that the interferometer signals can still be received while the sample is rotated in X-ray tomography experiments. In the current design using the half-coated sphere the sample can be rotated over an angular range of more than 180°. The interferometer heads are connected via 20 m long optical fibres to the interferometer controller (PicoScale, SmarAct GmbH). The working wavelength of the interferometer is 1530 nm. A visible-light alignment laser can be activated independently. The position acquisition is triggered synchronously with the area detectors and fluorescence pulse processors of the beamline and permits fast continuous scanning with image acquisition rates of the order of 1 kHz. As the maximum sampling frequency is 156 kHz, the sample motion during each exposure can be analysed individually even with such rapid data acquisition. Besides the sample displacement, the raw quadrature signal can also be saved for more detailed analysis. Positioning inaccuracies of the sample related to tilt errors introduced by the piezo scanner are not tracked with the current setup. They are specified by the manufacturer with a maximum error of 6 µrad, leading to a maximum induced parallax error in the range of 100 nm. Further instrumental improvements will be needed to measure and compensate for this effect.

## 4. X-ray optics and nanobeam characterization   

The core part of PtyNAMi is designed to accommodate different nanofocusing X-ray optics, such as NFLs (Schroer *et al.*, 2003 ▸, 2004 ▸, 2005 ▸) for the harder X-ray regime above *E* = 10 keV and FZPs (Vila-Comamala *et al.*, 2011 ▸; Gorelick *et al.*, 2011 ▸; Parfeniukas *et al.*, 2016 ▸; Mohacsi *et al.*, 2016 ▸) for the lower X-ray spectrum below *E* = 10 keV. The setup is quite flexible, providing a general platform for the characterization of new X-ray optics (Seiboth *et al.*, 2014 ▸; Lyubomirskiy *et al.*, 2019 ▸). In particular, adiabatically focusing lenses (AFLs) (Schroer & Lengeler, 2005 ▸; Patommel *et al.*, 2017 ▸) or multilayer Laue lenses (Kang *et al.*, 2008 ▸; Bajt *et al.*, 2018 ▸) can reach the sub-20 nm resolution regime on a routine basis.

**Figure 5:**
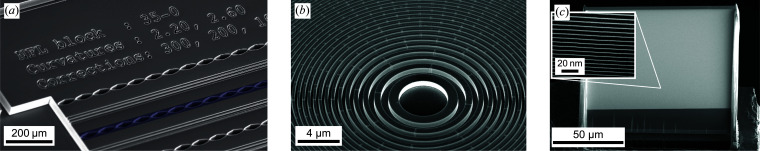
SEM images of (*a*) an NFL structured in silicon (Schroer *et al.*, 2005 ▸), (*b*) an FZP made out of gold with typical layer height from 0.85 to 1 µm and smallest outermost zone width of 50 nm (Gorelick *et al.*, 2011 ▸), and (*c*) an MLL (Bajt *et al.*, 2018 ▸).

Fig. 5 ▸ shows different high-performance X-ray optics typically implemented in PtyNAMi. In the case of cylindrical lenses focusing in one dimension only (1D focusing), such as NFLs or MLLs, two such lenses have to be implemented in a crossed geometry in order to create a 2D point focus. Each lens is then mounted on a hexapod to align the lens fully with six degrees of freedom, *i.e.* three translations and three rotations. At a distance further downstream a pinhole is implemented in order to reduce parasitic scattering created within the lenses or other upstream components. For the FZP, on the other hand, the lens is typically mounted on one of the lens hexapods, while the other one holds a beam stop to block the central unfocused part of the X-ray beam. In this case the pinhole acts as an order-sorting aperture, cleaning the nanofocused X-ray beam of other diffraction orders of the zone plate.

**Figure 6:**
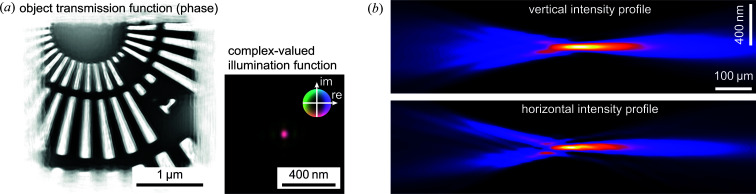
Characterization of a nanofocused X-ray beam by ptychography using the previous nanoprobe setup on beamline P06. (*a*) The illumination and object function were reconstructed using the ePIE algorithm. (*b*) Caustics of the nanofocused X-ray beam in the vertical and horizontal planes.

In Fig. 6 ▸ a typical result for nanobeam characterization by ptychography is shown, which was measured using the previous nanoprobe setup before the upgrade. Here, a resolution test chart by NTT-AT (model ATN/XRESO-50HC) made of tantalum with a material thickness of 500 nm was scanned over an area of 2 × 2 µm on a 2D grid with 50 × 50 steps. At each scan position, a far-field diffraction pattern was recorded with an exposure time of 0.5 s at an X-ray photon energy of *E* = 18 keV using a Pilatus detector (Dectris Ltd). The detector has a pixel size of *p* = 172 µm and was positioned at a distance of 2.24 m downstream of the sample. From the total of 2601 measured diffraction patterns, both the complex-valued transmission function of the sample and the illumination function can be recovered at the same time using *e.g.* the standard ePIE algorithm (Maiden & Rodenburg, 2009 ▸). In Fig. 6 ▸ the result of this beam-characterization experiment is summarized, showing the phase of the object transmission function and the illumination function recovered in the sample plane. The amplitude of the illuminating complex wavefield is encoded by brightness and the phase by hue (compare inset). Note the relatively strong smearing of the smallest features with a size of 50 nm, especially in the horizontal direction [see Fig. 6 ▸(*a*)], which was often observed with the old setup.

**Figure 7:**
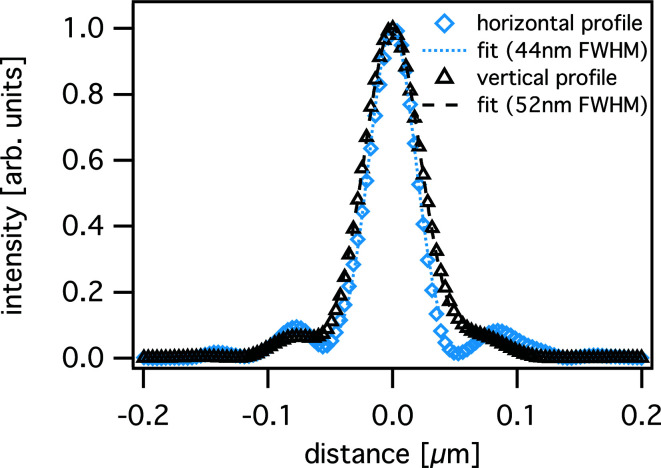
Horizontal and vertical intensity profiles in the focal plane obtained from the reconstructed illumination function shown in Fig. 6 ▸(*a*).

With the knowledge of the complex-valued illumination function, full information about the X-ray optics is obtained and the beam caustic can be retrieved by numerically propagating the wavefield along the X-ray beam path [see Fig. 6 ▸(*b*)], indicating that the X-ray focus is almost perfect with only low-intensity side lobes. The size of the nanofocus was determined to be 44 nm (h) × 52 nm (v) (see Fig. 7 ▸), only slightly larger than the theoretical diffraction limit of 40 nm (h) × 51 nm (v) for this lens configuration.

## 5. Mechanical stability and control   

**Figure 8:**
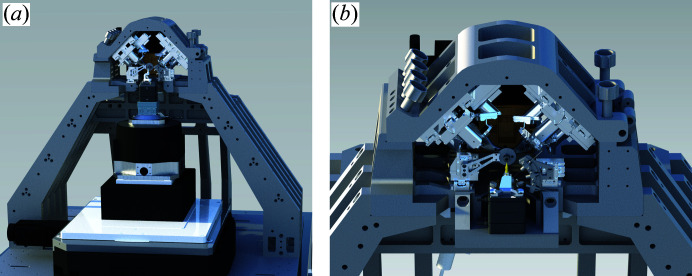
(*a*) The standard PtyNAMi setup, including all stages for coarse sample alignment and rotation. (*b*) In the ultrastable configuration of the setup, the piezo scanner is attached directly to the optics frame.

In a first step the mechanical setup of the microscope was considerably improved by stiffening the microscope frame using titanium struts and an Invar casing to reduce the effects of temperature drift. The construction was then optimized by finite element methods in order to reduce the amplitudes of vibrational modes as much as possible and push the eigenfrequencies as high as possible. The result of this process is the standard PtyNAMi setup as illustrated in Fig. 8 ▸(*a*). In this configuration, the setup provides the greatest flexibility to carry out 3D X-ray imaging experiments requiring all stages for coarse sample alignment, rotation and centring. In order to give further control over the position of the sample during the experiment, a set of three optical interferometers was implemented. In this way, sample drifts or image distortions related to inaccuracies of the high-precision piezo scanner can be corrected. In addition, position values can now be recorded at sampling rates considerably above the scanning frequency, revealing information on the vibration modes of the sample relative to the frame during the acquisition time of single exposures.

It has been observed that the stability of the microscope improves still further as soon as all stages for coarse sample positioning are removed. In this ‘ultrastable’ configuration of PtyNAMi, the piezo scanner is directly attached to the Invar frame of the optics [see Fig. 8 ▸(*b*)]. Here, the sample tower is substantially smaller, thereby reducing the influence of relative motions and instabilities between the X-ray lenses and the sample. Of course, in this configuration the setup is less flexible and only 2D imaging experiments on pre-aligned samples can be carried out. While long-term drifts can often be corrected numerically, the ultrastable configuration has the main advantage that the influence of mechanical vibrations during single exposures, which otherwise lead to an incoherent superposition of diffraction data, is reduced effectively.

**Figure 9:**
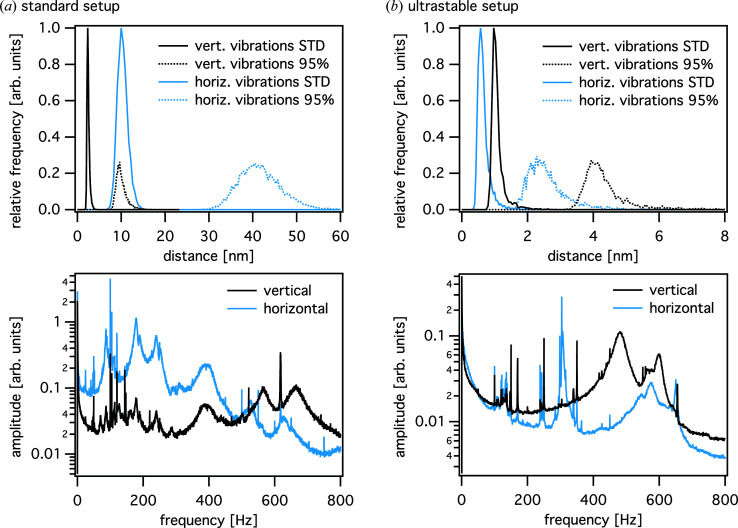
Upper plots: histograms of the standard deviation (STD) and the width of the distribution containing 95% of the position values, determined from the interferometer data during a typical 2D ptychographic scan in the case of (*a*) the standard tomographic setup and (*b*) the ultrastable configuration. Lower plots: frequency spectra obtained from the same scans. For the ultrastable configuration, the resonance frequencies are reduced by about an order of magnitude, especially in the lower-frequency regime. The frequency spectra use the same arbitrary units on the ordinate.

The stability of these two configurations of the setup can be assessed with the help of the interferometer data measured *e.g.* during typical step scans. Since these devices are operated at a high repetition rate, a large number of position values can be recorded for each scan point, which can then be further analysed by statistical means. In Fig. 9 ▸ the stability statistics are given for both the standard and the ultrastable setup. For each position in the scan, the centre of mass and the standard deviation of the point cloud are calculated. In addition, the width of the point cloud is determined that includes 95% of all points, discarding the outermost 2.5% of the points on each side of the distribution. Fig. 9 ▸ shows the histograms of the standard deviation and the 95% width in the vertical (black lines) and horizontal (light-blue lines) directions, respectively. In the standard setup, typical vibrational excursions lie in the range of tens of nanometres horizontally [Fig. 9 ▸(*a*)] and up to 10 nm vertically (95% width). In the ultrastable setup, the point cloud is significantly smaller, covering an area of 2 nm (h) × 4 nm (v) (95% width). Low-frequency vibrations are considerably reduced using the ultrastable setup, as can be recognized in the corresponding Fourier spectra (see lower graphs in Fig. 9 ▸).

In the standard setup, the relatively large vibrational level compared with that of the ultrastable setup can be attributed to the coarse sample stages. In the ultrastable setup, a high degree of stability is obtained at the expense of flexibility of the scanning modes, limiting applications to 2D scans of well pre-aligned samples. Currently, the sample scanner has been redesigned to combine the advantages of both setups, yielding high stability and allowing flexible alignment of more complicated sample environments and tomographic scanning modes.

**Figure 10:**
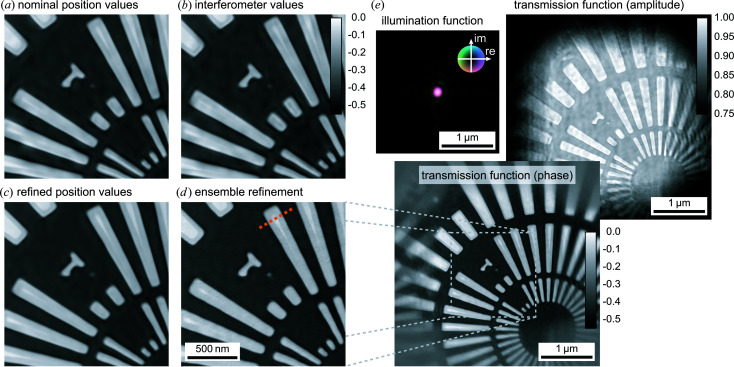
Results of a ptychographic imaging experiment demonstrating the high performance of the ultrastable configuration of PtyNAMi. Panels (*a*)–(*d*) illustrate the influence of the position values and mechanical instabilities on the ptychographic reconstruction result of the phase. (*e*) The final reconstruction result, showing the retrieved illumination function and the amplitude and phase of the object transmission function.

In the following, we illustrate high-resolution imaging using the ultrastable setup of PtyNAMi in view of positioning errors. In particular, different positioning data are used in the reconstruction, as well as different reconstruction models (Fig. 10 ▸).

**Figure 11:**
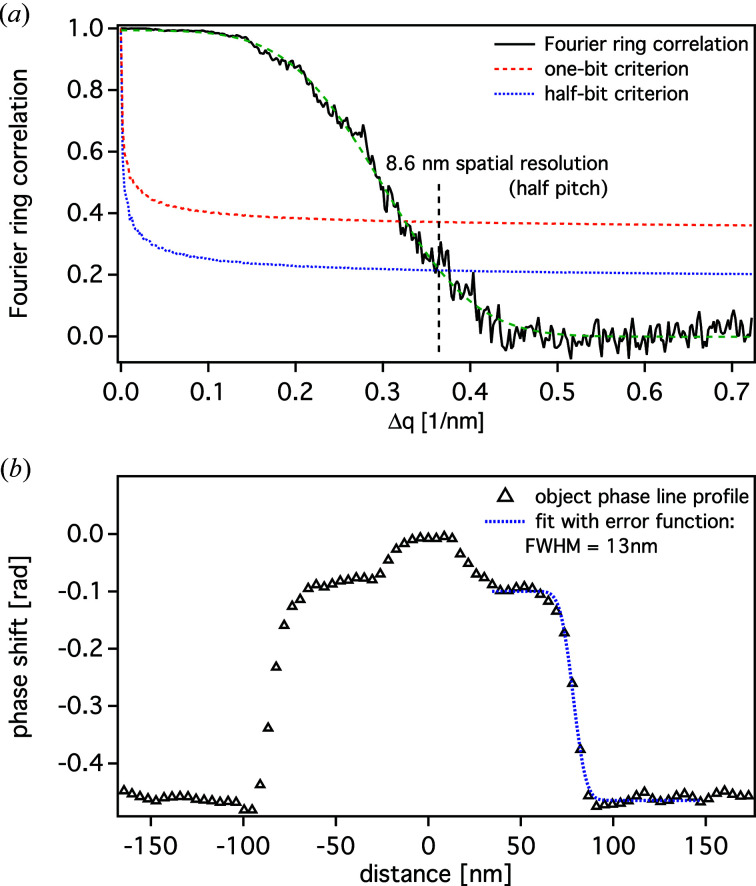
(*a*) The Fourier ring correlation calculated from Fig. 10 ▸(*d*). The half-bit criterion yields a half-pitch resolution of 8.6 nm. (*b*) The line profile extracted from the same image [*cf.* orange dashed line in Fig. 10 ▸(*d*)], yielding an edge width of about 13 nm (FWHM).

The imaging experiment was carried out on the same resolution test chart as shown in Fig. 6 ▸. In this case, an area of 2 × 2 µm was scanned on a 2D quadratic grid with 60 × 60 equidistant steps and an exposure time of 0.5 s per scan point. The result is summarized in Fig. 10 ▸, which shows different reconstruction runs using encoder position values [Fig. 10 ▸(*a*)] and values measured by the optical interferometers [Fig. 10 ▸(*b*)]. Also shown are ptychographic reconstructions starting with the interferometer positioning data, which are then iteratively refined using a brute-force local search approach [Fig. 10 ▸(*c*)], and the final result starting with the reconstruction result shown in Fig. 10 ▸(*c*) but additionally refined with one iteration using an algorithm further correcting incoherence effects related to sample vibrations during single exposures [Fig. 10 ▸(*d*)] (manuscript in preparation). It demonstrates a considerable improvement in image quality, showing that the finest structures of the resolution test chart of 50 nm are clearly resolved. In order to retrieve a value for the achieved spatial resolution, the images were evaluated by Fourier ring correlation (FRC) (van Heel & Schatz, 2005 ▸; Banterle *et al.*, 2013 ▸). For this calculation, the data set was split into two parts containing only half of the data points each, which was still sufficient for ptychographic phase retrieval. The two independently reconstructed images were then analysed by FRC, yielding a half-pitch spatial resolution of 8.6 nm if the half-bit criterion was applied [see Fig. 11 ▸(*a*)]. The FRC analysis showed only small deviations between the images presented in Figs. 10 ▸(*a*)–10 ▸(*d*), since mostly long-scale errors or distortions are corrected by using the interferometer data. In this case, the spatial resolution is mainly limited by the available coherent photon flux at the instrument. Additionally, an example line profile over a single bar of the resolution test chart was extracted, indicating an edge sharpness of approximately 13 nm [see Fig. 11 ▸(*b*)]. The position of the line profile is indicated by a dashed orange line in Fig. 10 ▸(*d*).

## 6. Signal-to-background optimization for high resolution and sensitivity   

In the standard ptychographic model, the far-field diffraction pattern is generated by scattering the probe beam off the sample. In a real ptychographic experiment, radiation from other sources can contribute to the measured signal, such as scattering from optical components, windows or residual gas along the beam path. The additional signal is interpreted by the algorithm as being part of the object, leading to reconstruction artefacts (Reinhardt *et al.*, 2017 ▸). While this additional background radiation can in principle be included in the ptychographic model (Bernert *et al.*, 2017 ▸), it can cover up the signal from more weakly scattering parts of the sample if, for example, the noise level of the background exceeds the signal level of the weakly scattering objects. This limits the sensitivity and resolution of the method (Schroer, Seyrich *et al.*, 2019 ▸).

**Figure 12:**
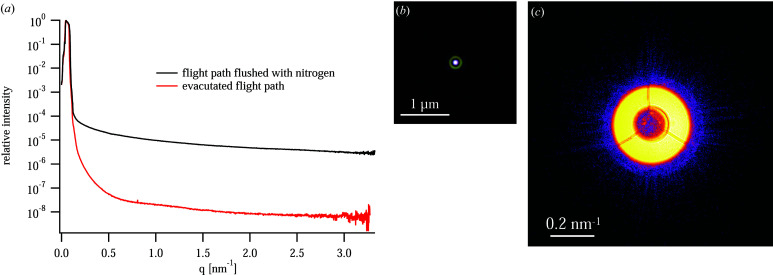
(*a*) The beam intensity on the detector without any sample in the beam: azimuthal average for the case where the flight tube is flushed with nitrogen (black curve) and the evacuated case (8 × 10^−4^ mbar, red curve). In this experiment, the coherent nanobeam was created by an FZP. (*b*) The complex wavefield in the focal plane of the FZP with a central stop, as determined by ptychography. (*c*) A far-field image of the nanobeam as measured on the pixel detector (cropped to 600 × 600 pixels around the optical axis). [Reprinted with permission from Schroer, Seyrich *et al.* (2019 ▸), copyright (2019) SPIE.]

To improve the sensitivity of PtyNAMi, the scattering background along the beam path behind the sample is minimized. This is achieved by mounting a windowless SAXS detector (Eiger X 4M) inside a vacuum tube (see Fig. 3 ▸). To keep the sample environment as flexible as possible, *e.g.* to accommodate special sample environments (see Section 7.2), the sample stage is in air. The only optical element between the sample and the detector is the entrance window at the tip of the nozzle very close to the sample and far away from the detector. The entrance window can be a single-crystal diamond (thickness 100 µm) or Kapton window (thickness 25 µm) to minimize the scattering from it. We typically prefer to use the diamond window since the small-angle X-ray scattering background is low for single-crystalline materials. However, the Kapton window performs similarly well in most cases because prominent Kapton scattering emerges at larger scattering angles beyond the small-angle regime covered in ptychography and therefore does not contribute to the background signal. Fig. 12 ▸(*c*) shows the far-field image on the detector of a nanobeam generated by an FZP at *E* = 9.3 keV [see Schroer, Seyrich *et al.* (2019 ▸) for details]. With the flight path flushed with nitrogen at ambient pressure, the relative intensity of the background in the far-field image of the nanobeam drops by about five orders of magnitude relative to the direct beam [see Fig. 12 ▸(*a*)], while with an evacuated flight path the background improves by three orders of magnitude to a level of 10^−8^ relative to the direct beam.

This is an appropriate level to image nanoparticles of a few tens of nanometres in size (Schroer, Seyrich *et al.*, 2019 ▸), but it will not be sufficient to image more weakly scattering single-digit-nanometre objects. The main factors contributing to this residual background need to be investigated further.

## 7. High-resolution X-ray microscopy on PtyNAMi   

In this section, we present a few representative results of nano-imaging experiments carried out on PtyNAMi. They highlight the different operation modes of the instrument and the scientific opportunities that arise from them. For example, by combining X-ray ptychography with scanning microscopy with various contrasts, the structure–function relationship of solar cells can be assessed directly (Section 7.1). Catalytic materials can be ptychographically imaged under *in situ* conditions, following processes with high spatial resolution at varying elevated temperatures (Section 7.2). Small catalytic particles of a few micrometres in diameter can be imaged with improved quality in three dimensions by direct coupling of ptychographic and tomographic methods (Section 7.3).

### 7.1. Multi-modal X-ray imaging   

**Figure 13:**
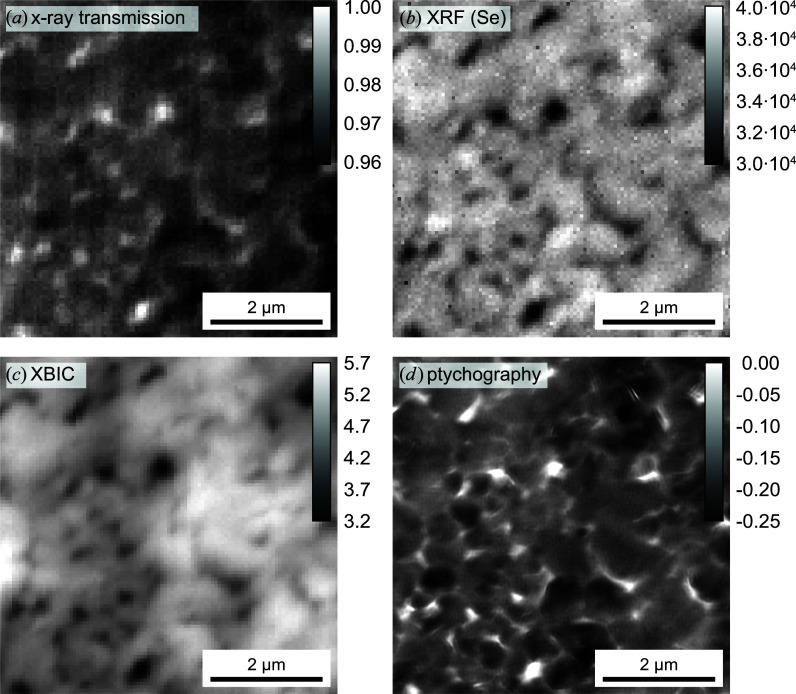
Multi-modal X-ray imaging of a solar cell with a CuIn_1−*x*_Ga_*x*_Se_2_ absorber layer. 2D maps of (*a*) the X-ray transmission signal (maximum intensity scaled to 1), (*b*) the XRF signal (sum of *K*α and *K*β) of selenium (counts per second), (*c*) the XBIC signal (nanoamperes) and (*d*) the ptychographically reconstructed phase of the object transmission function (radians). See also the greyscale bars in each image.

A main strength of scanning hard X-ray microscopy is that different X-ray analytical contrasts can be accessed all at once (Schropp *et al.*, 2011 ▸; Kahnt *et al.*, 2018 ▸; Falkenberg *et al.*, 2018 ▸; Stachnik *et al.*, 2020 ▸). This multi-modal approach allows one to investigate functional materials such as thin-film solar cells under different scientific perspectives and correlate, for example, their function with their elemental composition or structural properties (Stuckelberger *et al.*, 2017 ▸). Despite its strict stability requirements, PtyNAMi provides enough flexibility to carry out such experiments. Fig. 13 ▸ highlights the result of such an experiment that was carried out on a solar cell with a 2 µm thick CuIn_1−*x*_Ga_*x*_Se_2_ absorber layer on an X-ray-transparent substrate (Carron *et al.*, 2019 ▸). The sample was mounted perpendicular to the incident X-ray beam and scanned over an area of 6 × 6 µm on a 2D grid with 100 × 100 steps and an exposure time of 0.5 s per scan point. X-rays with an energy of *E* = 15.25 keV were focused onto the sample surface by a set of NFLs. The focus size was determined to be 105 nm (h) × 120 nm (v).

Here, ptychography was combined with X-ray fluorescence (XRF) and lock-in amplified X-ray beam-induced current (XBIC) measurements (Ossig *et al.*, 2019 ▸). This allowed the simultaneous assessment of the X-ray transmittance [Fig. 13 ▸(*a*)], the XRF signal of selenium (sum of *K*α and *K*β) [Fig. 13 ▸(*b*)], the XBIC signal [Fig. 13 ▸(*c*)] and the ptychographically reconstructed phase of the transmission function of the sample. For the ptychographic image, a spatial resolution of 39 nm was determined by FRC (see Section 5). Note that the ptychographic reconstruction did not suffer from significant artefacts, due to the X-ray chopper that was located in the microhutch and which modulated the X-ray beam at a frequency of 9763.6 Hz for the highest signal-to-noise ratio of the XBIC measurements. Beyond the intrinsic advantage of point-by-point correlation that is enabled by the simultaneous assessment of different modalities, the combination with ptychography offers the correction of imprecise position data for the co-measured XRF and XBIC data.

The (anti-)correlation of the four maps is striking and can easily be explained: the transmittance is a low-resolution equivalent to the phase shift representing the projected electron density in the solar-cell stack. In both maps, topological variations in the solar-cell absorber layer (Avancini *et al.*, 2018 ▸), represented here by the Se area density, dominate and govern the electrical performance that is evaluated as XBIC.

Of greater scientific interest are the deviations from this pattern. A detailed statistical analysis of the correlations, such as by Stuckelberger *et al.* (2020 ▸) or West *et al.* (2017 ▸), can provide insights into the recombination mechanisms of specific defect types, the impact of stoichiometric inhomogeneities or defect passivation at topological features such as voids.

### 7.2. *In situ* hard X-ray ptychography   

**Figure 14:**
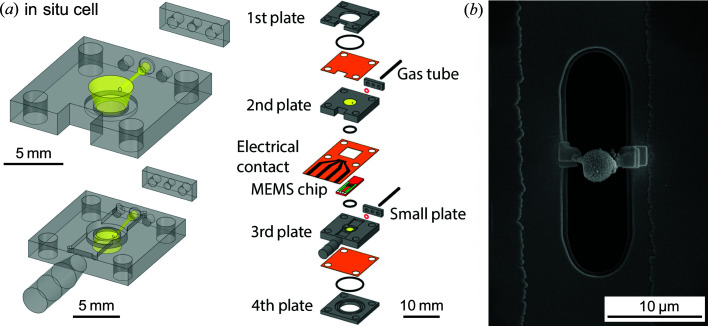
(*a*) A schematic diagram of the sample environment for *in situ* ptychography with a limited tilting angle, including the MEMS chip sample holder (Fam *et al.*, 2019 ▸). (*b*) A SEM image acquired during FIB–SEM showing a CoMn_2_O_4_ spinel ‘hollow-sphere’ particle of approximately 3 µm diameter attached to the viewing window of the MEMS chip.

In order to expand the experimental capabilities of PtyNAMi towards heterogeneous catalysis research, a series of *in situ* sample environments were developed for the instrument, described in detail in a previous publication by Fam *et al.* (2019 ▸). In summary, the *in situ* cells [see Fig. 14 ▸(*a*)] permit excellent control of environmental conditions, including localized temperature treatment up to above 1273 K with a stability of around ±0.1 K by resistive heating, and controlled gas environments up to 1 ml min^−1^ total flow. The sample holders utilize microelectromechanical systems (MEMS) chips (Wildfire, DENSsolutions BV) originally designed for electron microscopy and tomography, here repurposed for high-resolution X-ray nano-imaging. This allows for treatments such as oxidation and reduction, along with catalytic conditions at atmospheric pressure. The gas environment and potential products are monitored by mass spectrometry of the outlet gas stream.

Samples are directly loaded onto the MEMS chips via focused ion beam milling combined with scanning electron microscopy (FIB–SEM) [Fig. 14 ▸(*b*)], in this case performed at DESY NanoLab (Stierle *et al.*, 2016 ▸). Fig. 14 ▸(*b*) shows a sample of CoMn_2_O_4_ spinel with a hollow-sphere structure, consisting of a dense spinel core and a thin spinel shell of the same material, with an air buffer in between. Such samples are suggested as potential candidates for chemical conversions in confined environments, with the small air buffer and permeable exterior shell forming a small ‘nanoreactor’ (Arnal *et al.*, 2006 ▸; Sun *et al.*, 2013 ▸). Such hollow spheres also have potential applications in gas sensing (Li *et al.*, 2004 ▸). Due to their characteristic structure, these materials are a suitable case study to demonstrate *in situ* nano-imaging during thermal decomposition, with length-scale changes of the order of several hundreds of nanometres expected.

**Figure 15:**
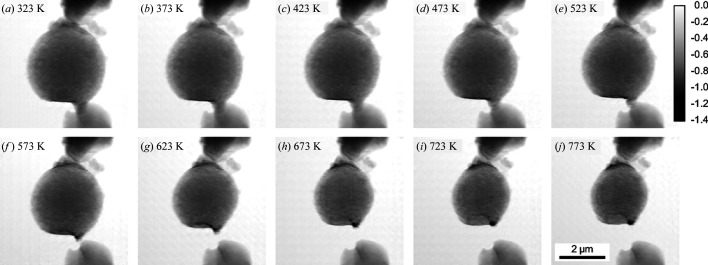
(*a*)–(*j*) A 2D ptychographic image series of the pre-calcined CoMn_2_O_4_ hollow sphere during heating in synthetic air from 323 to 773 K. The greyscale shows the phase shift in radians.

As shown in Fig. 15 ▸, a single hollow-sphere sample was imaged by ptychography in the *in situ* cell during sequential thermal treatment up to 773 K in synthetic air. Here, the experiment was carried out at an X-ray photon energy of *E* = 9 keV using an FZP as focusing optics, and a spatial resolution of about 20 nm was determined by FRC for this ptychographic *in situ* experiment (see Section 5). The *in situ* cells effectively enable the structural deactivation effects of catalytic materials to be monitored under precisely controlled conditions, and with sample sizes greatly exceeding those possible using transmission electron microscopy (TEM) or electron tomography. It should be noted that the MEMS chips can in fact be used directly for complementary TEM imaging, if the sample size permits. In addition, limited-angle rotation has shown potential for the acquisition of ptycho-tomographic data under *in situ* conditions, with the potential to examine 3D structural changes in greater detail (Yang *et al.*, 2020 ▸).

### 7.3. 3D ptychography   

Sample alignment on the nanometre scale is especially critical for ptychographic imaging in three dimensions, since many projections of a sample need to be recorded from different perspectives in order to finally extract volumetric information (Dierolf *et al.*, 2010 ▸). Due to the still limited coherent flux at third-generation X-ray sources, such a 3D data set is typically recorded over many hours, and sample vibrations, drifts or angular misalignments can considerably complicate the subsequent data analysis. Although some of these experimental inaccuracies, *e.g.* the mutual alignment of images, can be corrected quite well numerically, the stability of the setup is still important to obtain sharp and undistorted 2D projections, which are essential for optimal results in 3D ptychography.

**Figure 16:**
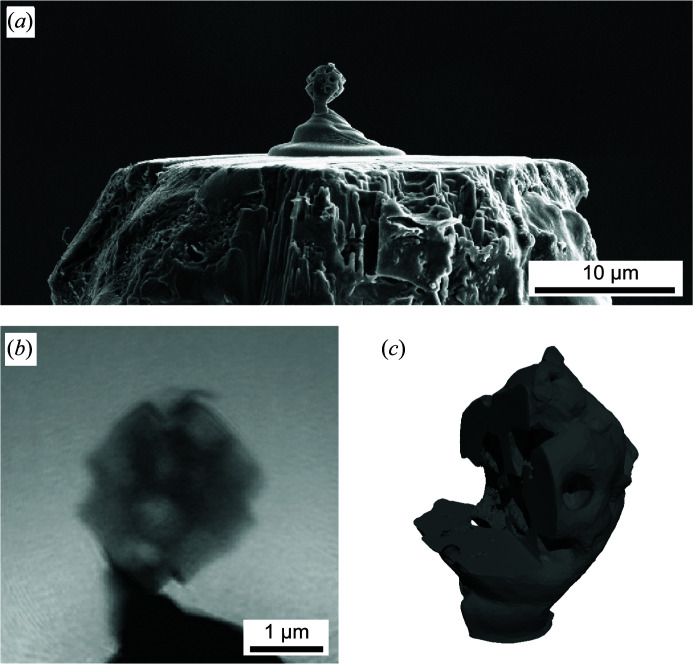
(*a*) A SEM image of a macroporous zeolite particle with a size of about 2.6 µm. It was glued to the tip of an aluminium pin by a platinum pedestal using FIB–SEM. (*b*) A 2D phase map of the reconstructed object transmission function. (*c*) A 3D isosurface rendering of the reconstructed volume (phase). The cutout reveals the inner pore structure of the sample [adapted from Kahnt *et al.* (2019 ▸); copyright 2019 Optical Society of America under the terms of the OSA Open Access Publishing Agreement].

In Fig. 16 ▸ the result of such a 3D ptychographic experiment carried out on PtyNAMi is summarized (Kahnt *et al.*, 2019 ▸). Similarly to the previous example, the experiment was carried out at an X-ray photon energy of *E* = 9 keV using an FZP as focusing optics to create a nanofocus with a size of about 70 nm (FWHM). In this case, a freestanding macroporous zeolite particle composed of porous silica and alumina [Fig. 16 ▸(*a*)] was raster-scanned through the coherent nanobeam at a distance of 1 mm behind the focal plane and over an area about 4 × 4 µm. Each 2D projection was then reconstructed from a set of 121 diffraction patterns (11 × 11 scan points), each exposed for 1 s. Altogether, 90 2D projections were captured over an angular range of 180°, requiring a total measurement time of about 7 h for the whole 3D ptychographic experiment. Fig. 16 ▸(*b*) shows an example of the reconstructed phase of the sample transmission function in a single 2D projection.

From this 3D data set the sample volume can be reconstructed, and an iso-surface rendering [Fig. 16 ▸(*c*)] clearly highlights the macroporous structure of the zeolite particle with a spatial resolution of about 65 nm. Additional detailed information on 3D ptychographic data evaluation and the achieved spatial resolution can be found in the report by Kahnt *et al.* (2019 ▸) and supplementary material therein. Furthermore, ptychographic and tomographic algorithms were directly coupled in this case, taking advantage of mitigating sampling or dose requirements in 3D ptychography. The direct tomographic reconstruction method may also facilitate the often time-consuming position refinement steps during data evaluation.

## 8. Summary and outlook   

Hard X-ray ptychography has developed considerably over the past decade and the method has matured to become a standard technique at various synchrotron radiation sources worldwide. Here, we have presented our efforts on beamline P06 to redesign and build a new generation of scanning X-ray microscopes by optimizing the instrument from the point of view of mechanical stability, positioning control and reduced background scattering. In the standard tomographic configuration a typical spatial resolution of a few tens of nanometres can be reached, depending on sample contrast and measurement time. Up to now, this has mainly been limited by vibrations introduced by the coarse alignment stages, which we aim to replace in the near future.

Hard X-ray nano-imaging at a spatial resolution of slightly below 10 nm could be demonstrated using an ultrastable configuration of the setup, but further improvements are necessary if the full flexibility of the setup, including sample rotation for tomographic applications or other auxiliary equipment, is required. We consider these instrumental improvements to be crucial in order to push the spatial resolution in hard X-ray microscopy towards the single-digit nanometre regime. These developments are driven strongly by upcoming fourth-generation X-ray sources, which will provide a considerably larger coherent photon flux, making these instruments excellent 3D microscopes to image structural, chemical or physical processes on all length scales down to the atomic scale.

## Figures and Tables

**Table 1:** Beamline components on P06 and their distance from the centre of the undulator [updated from (Schroer *et al.*, 2016 ▸)]

Beamline component	Position (m)
U32 undulator	0.00
Vertical high-power slit PS1	18.91
High-power slits PS2	26.69
Multilayer monochromator (MLM)	35.81
Si monochromators, DCM and CCM	38.40
Beam position monitor QBPM mono	39.34
Retractable screen LM2	39.51
First HO mirror	40.78
Second HO mirror	41.78
Refractive X-ray lenses (Si lenses)	42.61
Retractable screen LM2b	42.69
Optical hutch (OH) slits	42.95
	43.35
Refractive X-ray lenses CRLs	43.54
Retractable screen LM3	44.66
Retractable screen LM4	86.66
Beam position monitor QBPM micro	86.83
Fast shutter	87.39
Absorber unit	87.75
Scanner unit entrance slits	97.35

**Table 2:** The prefocusing system on beamline P06 provides space for six individual Be CRL cartridges, each containing an individual lens configuration defined by the number *N* of CRLs with a specific radius of curvature *R*

Cartridge No.	*R* = 1.5 mm	*R* = 500 µm	*R* = 200 µm
1	*N* = 2		
2	*N* = 1	*N* = 1	
3	*N* = 2	*N* = 2	
4	*N* = 1	*N* = 5	
5	*N* = 2	*N* = 10	
6			*N* = 9

**Table 3:** PtyNAMi components and their distance from the beam-defining slits Given values refer to a set of NFLs with a working distance of 30 mm.

PtyNAMi component	Position (mm)
Entrance slits	0.0
Transmission diode	43.0
Vertical focusing NFL	91.8
Horizontal focusing NFL	104.9
Pinhole	122.6
Sample	134.0

## References

[bb1] Arnal, P. M., Comotti, M. & Schüth, F. (2006). *Angew. Chem. Int. Ed.* **45**, 8224–8227. 10.1002/anie.200603507 17109458

[bb2] Avancini, E., Keller, D., Carron, R., Arroyo-Rojas Dasilva, Y., Erni, R., Priebe, A., Di Napoli, S., Carrisi, M., Sozzi, G., Menozzi, R., Fu, F., Buecheler, S. & Tiwari, A. N. (2018). *Sci. Technol. Adv. Mater.* **19**, 871–882. 10.1080/14686996.2018.1536679 PMC6249540 30479675

[bb3] Bajt, S., Prasciolu, M., Fleckenstein, H., Domaracky, M., Chapman, H. N., Morgan, A. J., Yefanov, O., Messerschmidt, M., Du, Y., Murray, K. T., Mariani, V., Kuhn, M., Aplin, S., Pande, K., Villanueva-Perez, P., Stachnik, K., Chen, J. P. J., Andrejczuk, A., Meents, A., Burkhardt, A., Pennicard, D., Huang, X., Yan, H., Nazaretski, E., Chu, Y. S. & Hamm, C. E. (2018). *Light Sci. Appl.* **7**, 17162. 10.1038/lsa.2017.162 PMC6060042 30839543

[bb4] Banterle, N., Bui, K. H., Lemke, E. A. & Beck, M. (2013). *J. Struct. Biol.* **183**, 363–367. 10.1016/j.jsb.2013.05.004 23684965

[bb5] Bernert, C., Hoppe, R., Wittwer, F., Woike, T. & Schroer, C. G. (2017). *Opt. Express*, **25**, 31640. 10.1364/OE.25.031640 29245835

[bb6] Boesenberg, U., Ryan, C. G., Kirkham, R., Siddons, D. P., Alfeld, M., Garrevoet, J., Núñez, T., Claussen, T., Kracht, T. & Falkenberg, G. (2016). *J. Synchrotron Rad.* **23**, 1550–1560. 10.1107/S1600577516015289 27787262

[bb7] Carron, R., Nishiwaki, S., Feurer, T., Hertwig, R., Avancini, E., Löckinger, J., Yang, S., Buecheler, S. & Tiwari, A. N. (2019). *Adv. Energy Mater.* **9**, 1900408.

[bb8] Deng, J., Preissner, C., Klug, J. A., Mashrafi, S., Roehrig, C., Jiang, Y., Yao, Y., Wojcik, M., Wyman, M. D., Vine, D., Yue, K., Chen, S., Mooney, T., Wang, M., Feng, Z., Jin, D., Cai, Z., Lai, B. & Vogt, S. (2019). *Rev. Sci. Instrum.* **90**, 083701. 10.1063/1.5103173 31472643

[bb9] Dierolf, M., Menzel, A., Thibault, P., Schneider, P., Kewish, C. M., Wepf, R., Bunk, O. & Pfeiffer, F. (2010). *Nature*, **467**, 436–439. 10.1038/nature09419 20864997

[bb10] Dzhigaev, D., Shabalin, A., Stankevič, T., Lorenz, U., Kurta, R. P., Seiboth, F., Wallentin, J., Singer, A., Lazarev, S., Yefanov, O. M., Borgström, M., Strikhanov, M. N., Samuelson, L., Falkenberg, G., Schroer, C. G., Mikkelsen, A., Feidenhans’l, R. & Vartanyants, I. A. (2016). *J. Opt.* **18**, 064007.

[bb70] Falkenberg, G., Fleissner, G., Alraun, P., Reinhardt, J., Scholz, M., Schropp, A., Spiers, K., Garrevoet, J., Schroer, C. G. & Fleissner, G. (2018). *J. Instrum.* **13**, C07001.

[bb11] Fam, Y., Sheppard, T. L., Becher, J., Scherhaufer, D., Lambach, H., Kulkarni, S., Keller, T. F., Wittstock, A., Wittwer, F., Seyrich, M., Brueckner, D., Kahnt, M., Yang, X., Schropp, A., Stierle, A., Schroer, C. G. & Grunwaldt, J.-D. (2019). *J. Synchrotron Rad.* **26**, 1769–1781. 10.1107/S160057751900660X PMC6732905 31490169

[bb12] Faulkner, H. M. L. & Rodenburg, J. M. (2004). *Phys. Rev. Lett.* **93**, 023903. 10.1103/PhysRevLett.93.023903 15323918

[bb13] Gorelick, S., Vila-Comamala, J., Guzenko, V. A., Barrett, R., Salomé, M. & David, C. (2011). *J. Synchrotron Rad.* **18**, 442–446. 10.1107/S0909049511002366 PMC3133522 21525653

[bb14] Heel, M. van & Schatz, M. (2005). *J. Struct. Biol.* **151**, 250–262. 10.1016/j.jsb.2005.05.009 16125414

[bb15] Holler, M., Raabe, J., Diaz, A., Guizar-Sicairos, M., Wepf, R., Odstrcil, M., Shaik, F. R., Panneels, V., Menzel, A., Sarafimov, B., Maag, S., Wang, X., Thominet, V., Walther, H., Lachat, T., Vitins, M. & Bunk, O. (2018). *Rev. Sci. Instrum.* **89**, 043706. 10.1063/1.5020247 29716370

[bb16] Hruszkewycz, S. O., Allain, M., Holt, M. V., Murray, C. E., Holt, J. R., Fuoss, P. H. & Chamard, V. (2017). *Nat. Mater.* **16**, 244–251. 10.1038/nmat4798 27869823

[bb17] Kahnt, M., Becher, J., Bückner, D., Fam, Y., Sheppard, T., Weissenberger, T., Wittwer, F., Grunwaldt, J.-D., Schwieger, W. & Schroer, C. G. (2019). *Optica*, **6**, 1282–1289.

[bb18] Kahnt, M., Falkenberg, G., Garrevoet, J., Hartmann, J., Krause, T., Niehle, M., Scholz, M., Seyrich, M., Trampert, A., Waag, A., Wehmann, H.-H., Wittwer, F., Zhou, H., Hanke, M. & Schroer, C. G. (2018). *Microsc. Microanal.* **24**, 32–33.

[bb19] Kang, H. C., Yan, H., Winarski, R. P., Holt, M. V., Maser, J., Liu, C., Conley, R., Vogt, S., Macrander, A. T. & Stephenson, G. B. (2008). *Appl. Phys. Lett.* **92**, 221114.

[bb20] Kirkpatrick, P. & Baez, A. (1948). *J. Opt. Soc. Am.* **38**, 766–774. 10.1364/josa.38.000766 18883922

[bb21] Lengeler, B., Schroer, C. G., Richwin, M., Tümmler, J., Drakopoulos, M., Snigirev, A. & Snigireva, I. (1999). *Appl. Phys. Lett.* **74**, 3924–3926.

[bb22] Li, X.-L., Lou, T.-J., Sun, X.-M. & Li, Y.-D. (2004). *Inorg. Chem.* **43**, 5442–5449. 10.1021/ic049522w 15310226

[bb23] Lyubomirskiy, M., Koch, F., Abrashitova, K. A., Bessonov, V. O., Kokareva, N., Petrov, A., Seiboth, F., Wittwer, F., Kahnt, M., Seyrich, M., Fedyanin, A. A., David, C. & Schroer, C. G. (2019). *Opt. Express*, **27**, 8639–8650. 10.1364/OE.27.008639 31052678

[bb24] Maiden, A. M. & Rodenburg, J. M. (2009). *Ultramicroscopy*, **109**, 1256–1262. 10.1016/j.ultramic.2009.05.012 19541420

[bb25] Martínez-Criado, G., Villanova, J., Tucoulou, R., Salomon, D., Suuronen, J.-P., Labouré, S., Guilloud, C., Valls, V., Barrett, R., Gagliardini, E., Dabin, Y., Baker, R., Bohic, S., Cohen, C. & Morse, J. (2016). *J. Synchrotron Rad.* **23**, 344–352. 10.1107/S1600577515019839 PMC5297598 26698084

[bb26] Mimura, H., Yumoto, H., Matsuyama, S., Sano, Y., Yamamura, K., Mori, Y., Yabashi, M., Nishino, Y., Tamasaku, K., Ishikawa, T. & Yamauchi, K. (2007). *Appl. Phys. Lett.* **90**, 051903.

[bb27] Mohacsi, I., Vartiainen, I., Guizar-Sicairos, M., Karvinen, P., Guzenko, V. A., Müller, E., Kewish, C. M., Somogyi, A. & David, C. (2016). *Opt. Lett.* **41**, 281–284. 10.1364/OL.41.000281 26766694

[bb28] Nazaretski, E., Yan, H., Lauer, K., Bouet, N., Huang, X., Xu, W., Zhou, J., Shu, D., Hwu, Y. & Chu, Y. S. (2017). *J. Synchrotron Rad.* **24**, 1113–1119. 10.1107/S1600577517011183 29091054

[bb29] Ossig, C., Nietzold, T., West, B., Bertoni, M., Falkenberg, G., Schroer, C. G. & Stuckelberger, M. E. (2019). *J. Vis. Exp.* **150**, e60001. 10.3791/60001 31498310

[bb30] Parfeniukas, K., Rahomäki, J., Giakoumidis, S., Seiboth, F., Wittwer, F., Schroer, C. G. & Vogt, U. (2016). *Microelectron. Eng.* **152**, 6–9.

[bb31] Patommel, J., Klare, S., Hoppe, R., Ritter, S., Samberg, D., Wittwer, F., Jahn, A., Richter, K., Wenzel, C., Bartha, J. W., Scholz, M., Seiboth, F., Boesenberg, U., Falkenberg, G. & Schroer, C. G. (2017). *Appl. Phys. Lett.* **110**, 101103.

[bb32] Pfeiffer, F. (2018). *Nat. Photon.* **12**, 9–17.

[bb33] Raimondi, P. (2016). *Synchrotron Rad. News*, **29**(6), 8–15.

[bb34] Reinhardt, J., Hoppe, R., Hofmann, G., Damsgaard, C. D., Patommel, J., Baumbach, C., Baier, S., Rochet, A., Grunwaldt, J.-D., Falkenberg, G. & Schroer, C. G. (2017). *Ultramicroscopy*, **173**, 52–57. 10.1016/j.ultramic.2016.11.005 27912167

[bb35] Rodenburg, J. M. & Faulkner, H. M. L. (2004). *Appl. Phys. Lett.* **85**, 4795–4797.

[bb36] Rodenburg, J. M., Hurst, A. C., Cullis, A. G., Dobson, B. R., Pfeiffer, F., Bunk, O., David, C., Jefimovs, K. & Johnson, I. (2007). *Phys. Rev. Lett.* **98**, 034801. 10.1103/PhysRevLett.98.034801 17358687

[bb37] Rumancev, C., Gräfenstein, A., Vöpel, T., Stuhr, S., von Gundlach, A. R., Senkbeil, T., Garrevoet, J., Jolmes, L., König, B., Falkenberg, G., Ebbinghaus, S., Schroeder, W. H. & Rosenhahn, A. (2020). *J. Synchrotron Rad.* **27**, 60–66. 10.1107/S1600577519014048 PMC6927521 31868737

[bb38] Schroer, C. G., Agapov, I., Brefeld, W., Brinkmann, R., Chae, Y.-C., Chao, H.-C., Eriksson, M., Keil, J., Nuel Gavaldà, X., Röhlsberger, R., Seeck, O. H., Sprung, M., Tischer, M., Wanzenberg, R. & Weckert, E. (2018). *J. Synchrotron Rad.* **25**, 1277–1290. 10.1107/S1600577518008858 PMC6140396 30179167

[bb39] Schroer, C. G., Baumbach, C., Döhrmann, R., Klare, S., Hoppe, R., Kahnt, M., Patommel, J., Reinhardt, J., Ritter, S., Samberg, D., Scholz, M., Schropp, A., Seiboth, F., Seyrich, M., Wittwer, F. & Falkenberg, G. (2016). *AIP Conf. Proc.* **1741**, 030007.

[bb40] Schroer, C. G., Boye, P., Feldkamp, J. M., Patommel, J., Samberg, D., Schropp, A., Schwab, A., Stephan, S., Falkenberg, G., Wellenreuther, G. & Reimers, N. (2010). *Nucl. Instrum. Methods Phys. Res. A*, **616**, 93–97.

[bb41] Schroer, C. G. & Falkenberg, G. (2014). *J. Synchrotron Rad.* **21**, 996–1005. 10.1107/S1600577514016269 PMC4151680 25177988

[bb42] Schroer, C. G., Kuhlmann, M., Hunger, U. T., Günzler, T. F., Kurapova, O., Feste, S., Frehse, F., Lengeler, B., Drakopoulos, M., Somogyi, A., Simionovici, A. S., Snigirev, A., Snigireva, I., Schug, C. & Schröder, W. H. (2003). *Appl. Phys. Lett.* **82**, 1485–1487.

[bb43] Schroer, C. G., Kuhlmann, M., Kurapova, O., Hunger, U. T., Günzler, T. F., Feste, S., Lengeler, B., Ziegler, S., Drakopoulos, M., Burghammer, M., Riekel, C., Snigirev, A. A. & Snigireva, I. I. (2004). *Proc. SPIE*, **5539**, 10–19.

[bb44] Schroer, C. G., Kurapova, O., Patommel, J., Boye, P., Feldkamp, J., Lengeler, B., Burghammer, M., Riekel, C., Vincze, L., van der Hart, A. & Küchler, M. (2005). *Appl. Phys. Lett.* **87**, 124103.

[bb45] Schroer, C. G. & Lengeler, B. (2005). *Phys. Rev. Lett.* **94**, 054802. 10.1103/PhysRevLett.94.054802 15783651

[bb46] Schroer, C. G., Röhlsberger, R., Weckert, E., Wanzenberg, R., Agapov, I., Brinkmann, R. & Leemans, W. (2019). *PETRA IV: Upgrade of PETRA III to the Ultimate 3DX-ray Microscope – Conceptual Design Report (CDR).* Hamburg: DESY.

[bb47] Schroer, C. G., Seyrich, M., Kahnt, M., Botta, S., Döhrmann, R., Falkenberg, G., Garrevoet, J., Lyubomirskiy, M., Scholz, M., Schropp, A. & Wittwer, F. (2017). *Proc. SPIE*, **10389**, 103890E.

[bb48] Schroer, C. G., Seyrich, M., Schropp, A., Döhrmann, R., Botta, S., Wiljes, P., Bückner, D., Kahnt, M., Wittwer, F., Grote, L., Koziej, D., Garrevoet, J. & Falkenberg, G. (2019). *Proc. SPIE*, **11112**, 111120D.

[bb49] Schropp, A., Boye, P., Goldschmidt, A., Hönig, S., Hoppe, R., Patommel, J., Rakete, C., Samberg, D., Stephan, S., Schöder, S., Burghammer, M. & Schroer, C. G. (2011). *J. Microsc.* **241**, 9–12. 10.1111/j.1365-2818.2010.03453.x 21118244

[bb50] Schropp, A., Brückner, D., Bulda, J., Falkenberg, G., Garrevoet, J., Seiboth, F., Wittwer, F., Koch, F., David, C. & Schroer, C. G. (2018). *Microsc. Microanal.* **24**, 186–187.

[bb51] Schropp, A., Hoppe, R., Patommel, J., Samberg, D., Seiboth, F., Stephan, S., Wellenreuther, G., Falkenberg, G. & Schroer, C. G. (2012). *Appl. Phys. Lett.* **100**, 253112.

[bb52] Schropp, A. & Schroer, C. G. (2010). *New J. Phys.* **12**, 035016.

[bb53] Seiboth, F., Scholz, M., Patommel, J., Hoppe, R., Wittwer, F., Reinhardt, J., Seidel, J., Knaut, M., Jahn, A., Richter, K., Bartha, J. W., Falkenberg, G. & Schroer, C. G. (2014). *Appl. Phys. Lett.* **105**, 131110.

[bb54] Seiboth, F., Schropp, A., Scholz, M., Wittwer, F., Rödel, C., Wünsche, M., Ullsperger, T., Nolte, S., Rahomäki, J., Parfeniukas, K., Giakoumidis, S., Vogt, U., Wagner, U., Rau, C., Boesenberg, U., Garrevoet, J., Falkenberg, G., Galtier, E. C., Ja Lee, H., Nagler, B. & Schroer, C. G. (2017). *Nat. Commun.* **8**, 14623. 10.1038/ncomms14623 PMC5337966 28248317

[bb55] Silva, J. C. da, Guilloud, C., Hignette, O., Jarnias, C., Ponchut, C., Ruat, M., Labiche, J.-C., Pacureanu, A., Yang, Y., Salome, M., Bohic, S. & Cloetens, P. (2019). *J. Synchrotron Rad.* **26**, 1751–1762. 10.1107/S1600577519006301 31490167

[bb56] Stachnik, K., Warmer, M., Mohacsi, I., Hennicke, V., Fischer, P., Meyer, J., Spitzbart, T., Barthelmess, M., Eich, J., David, C., Feldmann, C., Busse, B., Jähn, K., Schaible, U. E. & Meents, A. (2020). *Sci. Rep.* **10**, 1784. 10.1038/s41598-020-58318-7 PMC7000813 32019946

[bb57] Stankevič, T., Dzhigaev, D., Bi, Z., Rose, M., Shabalin, A., Reinhardt, J., Mikkelsen, A., Samuelson, L., Falkenberg, G., Vartanyants, I. A. & Feidenhans’l, R. (2015). *Appl. Phys. Lett.* **107**, 103101. 10.1021/acsnano.6b08122 28264155

[bb58] Stierle, A., Keller, T. F., Noei, H., Vonk, V. & Röhlsberger, R. (2016). *J. Large-Scale Res. Facil.* **2**, A76.

[bb60] Stuckelberger, M. E., Nietzold, T., West, B. M., Farshchi, R., Poplavskyy, D., Bailey, J., Lai, B., Maser, J. M. & Bertoni, M. I. (2020). *J. Phys. Energy*, **2**, 025001.

[bb59] Stuckelberger, M., West, B., Nietzold, T., Lai, B., Maser, J. M., Rose, V. & Bertoni, M. I. (2017). *J. Mater. Res.* **32**, 1825–1854.

[bb61] Sun, J., Xing, C., Xu, H., Meng, F., Yoneyama, Y. & Tsubaki, N. (2013). *J. Mater. Chem. A*, **1**, 5670.

[bb62] Takahashi, Y., Suzuki, A., Zettsu, N., Kohmura, Y., Senba, Y., Ohashi, H., Yamauchi, K. & Ishikawa, T. (2011). *Phys. Rev. B*, **83**, 214109.

[bb63] Tavares, P. F., Al-Dmour, E., Andersson, Å., Cullinan, F., Jensen, B. N., Olsson, D., Olsson, D. K., Sjöström, M., Tarawneh, H., Thorin, S. & Vorozhtsov, A. (2018). *J. Synchrotron Rad.* **25**, 1291–1316. 10.1107/S1600577518008111 PMC6140400 30179168

[bb64] Thibault, P., Dierolf, M., Menzel, A., Bunk, O., David, C. & Pfeiffer, F. (2008). *Science*, **321**, 379–382. 10.1126/science.1158573 18635796

[bb65] Vila-Comamala, J., Gorelick, S., Färm, E., Kewish, C. M., Diaz, A., Barrett, R., Guzenko, V. A., Ritala, M. & David, C. (2011). *Opt. Express*, **19**, 175–184. 10.1364/OE.19.000175 21263555

[bb66] West, B., Stuckelberger, M., Guthrey, H., Chen, L., Lai, B., Maser, J., Rose, V., Shafarman, W., Al-Jassim, M. & Bertoni, M. I. (2017). *Nano Energy*, **32**, 488–493.

[bb67] Yang, X., Kahnt, M., Brückner, D., Schropp, A., Fam, Y., Becher, J., Grunwaldt, J.-D., Sheppard, T. L. & Schroer, C. G. (2020). *J. Synchrotron Rad.* **27**, 486–493. 10.1107/S1600577520000831 PMC7064113 32153289

